# Organizational supports for knowledge translation in paediatric health centres and research institutes: insights from a Canadian environmental scan

**DOI:** 10.1186/s43058-021-00152-7

**Published:** 2021-05-13

**Authors:** Stephanie Miranda Nadine Glegg, Andrea Ryce, Kimberly J. Miller, Laura Nimmon, Anita Kothari, Liisa Holsti

**Affiliations:** 1 Rehabilitation Sciences, The University of British Columbia, T325 2211 Wesbrook Mall, Vancouver, BC V6T 2B5 Canada; 2 Sunny Hill Health Centre at BC Children’s Hospital, 4500 Oak Street, Vancouver, BC V6H 3N1 Canada; 3 BC Children’s Hospital Research Institute, 938 W. 28th Ave, Vancouver, BC V5Z 4H4 Canada; 4 Department of Occupational Science and Occupational Therapy, The University of British Columbia, T325 2211 Wesbrook Mall, Vancouver, BC V6T 2B5 Canada; 5 School of Health Studies, Western University, 1151 Richmond Street, London, Ontario N6A 3K7 Canada

**Keywords:** Organizational supports, Knowledge translation, Evidence-informed health care, Infrastructure, environmental scan

## Abstract

**Background:**

Organizational supports are thought to help address wide-ranging barriers to evidence-informed health care (EIHC) and knowledge translation (KT). However, little is known about the nature of the resources and services that exist within paediatric health care and research settings across Canada to facilitate evidence use in health care delivery. This survey examined existing supports for EIHC/KT within these organizations to inform the design of similar EIHC/KT support programmes.

**Methods:**

A national environmental scan was conducted using a bilingual online survey distributed to leaders at Canadian paediatric academic health science centres and their affiliated research institutes. Participants were invited through email, social media and webinar invitations and snowball sampling. Supports of interest included personnel, resources, services, organizational structures or processes, and partnerships or collaborations; barriers and successes were also probed. Data were compiled by site, reported using descriptive statistics, or grouped thematically. Supports were described using the AIMD (Aims, Ingredients, Mechanism, Delivery) framework.

**Results:**

Thirty-one respondents from 17 sites across seven provinces represented a 49% site response rate. Eleven (65%) sites reported an on-site library with variable staffing and services. Ten (59%) sites reported a dedicated KT support unit or staff person. Supports ranged from education, resource development and consultation to protocol development, funded initiatives and collaborations. Organizations leveraged internal and external supports, with the majority also employing supports for clinical research integration. Supports perceived as most effective included personnel, targeted initiatives, leadership, interdepartmental expertise, external drivers and logistical support. Barriers included operational constraints, individual-level factors and lack of infrastructure.

**Conclusions:**

This first survey of organizational supports for EIHC/KT identified the range of supports in place in paediatric research and health care organizations across Canada. The diversity of supports reported across sites may reflect differences in resource capacity and objectives. Similarities in EIHC/KT and research integration supports suggest common infrastructure may be feasible. Moreover, stakeholder engagement in research was common, but not pervasive. Tailored support programmes can target multi-faceted barriers. Findings can inform the development, refinement and evaluation of EIHC/KT support programmes and guide the study of the effectiveness and sustainability of these strategies.

**Supplementary Information:**

The online version contains supplementary material available at 10.1186/s43058-021-00152-7.

Contributions to the literature

- This article reports on the first national survey of organizational supports for knowledge translation, clinical research integration and stakeholder engagement in Canada
- This inventory of existing supports within academic health science centres and their affiliated research institutes can inform the design and refinement of organizational strategies to facilitate moving evidence into practice, policy and health service delivery
- The variability of supports suggests no one-size-fits-all approach exists; the categorization of these supports according to the AIMD framework supports the tailored selection of relevant strategies for different contexts.

## Background

Moving evidence into action is critical for ensuring safe, quality health service delivery that optimizes health outcomes [1, 2]. Evidence may include research findings, professional experience and patient/family perspectives, which are often considered together in making informed decisions about health care delivery [3, 4]. Evidence-informed health care (EIHC) is typically carried out by health care providers, health leaders and policy makers during the evidence-seeking, decision-making and implementation processes involved in developing, delivering and making changes to health services [5, 6]. Knowledge translation (KT) is usually understood to reflect the processes carried out or facilitated by researchers and others to identify the need for research evidence for decision-making, to effectively adapt/package and share evidence, to identify and apply strategies to address barriers to evidence implementation, and to evaluate evidence use in health care [7]. The EIHC and KT processes intersect at the end-users of the evidence. The implementation of supports for EIHC and KT within health centres, and other organizations engaged with them, has the potential to facilitate evidence use.

Limited resources (e.g. funding, staff), time constraints and negative attitudes toward change are among the primary barriers to organizational EIHC [8]. Organizational commitments, an EIHC culture, infrastructure to ensure access to evidence, EIHC competencies and linkages to expertise (e.g. researchers, knowledge brokers, librarians, others) are considered foundational for enabling, sustaining and evaluating consistent organizational EIHC [9, 10]. Appropriate investment in both human and financial resources are thought to produce the best chance for successful organizational support of KT [8]. However, no empirical evidence exists on the effectiveness of different types of supports to inform their prioritization [9, 10].

Organizational supports are being used increasingly to address the challenges health systems face in moving evidence into action [5, 10], but easily identifying these supports across the KT literature is difficult. To our knowledge, no organizational surveys on the topic have been published. A single Canadian study protocol from 2014 provides preliminary directions for conducting such a survey [10]; the findings of this study were not published because of a low response rate (M. Ouimet, personal communication, September 14, 2018). A second U.S. study protocol in the child and family services sector described aims to examine a limited number of KT support strategies (i.e. linkages to external knowledge brokers, technology infrastructure, personnel and training and organizational strategic planning [11]; however, results have not been published. An environmental scan can generate an overview of existing organization-based EIHC and KT supports. These findings can guide administrators to develop or refine programmes to facilitate evidence use. The insights have the potential to identify feasible support strategies and their key active ingredients, to inform their prioritization based on the identified needs, barriers to KT and available resources of an organization, and to highlight known gaps.

An environmental scan is a systematic and objective method of reviewing research evidence, stakeholder perspectives and current and anticipated policies, practices, processes and/or protocols across sectors [12, 13]. This data gathering methodology is used commonly within the business, policy and health care sectors to inform decision-making, to identify trends and promising practices, to avoid pitfalls experienced elsewhere and to understand the broader context outside one’s own organization [12–14]. The increasing prevalence of environmental scans is illustrated by examples, such as a scan of breastfeeding resources in Canadian neonatal intensive care units [15], of emergency response systems and services in remote First Nations communities [16], of local resources and needs related to vaccination guideline implementation [12], and of the evaluation of cancer genetics services across health care sites [14]. Research and grey literature searches are often combined with stakeholder engagement methods (e.g. surveys, interviews) to report on topics of interest across sectors [13]. This mixed methods approach is recognized as an efficient means of accounting for varied yet valuable sources of information, including tacit knowledge [13, 17]. The outcomes of the scan can be used to inform the design or strategic direction of projects or programmes [13, 14]. The purpose of this environmental scan was to identify existing organizational supports for EIHC/KT, clinical research integration and stakeholder engagement in research that exist within paediatric academic health science centres (AHSCs) and their affiliated research institutes across Canada. This article reports on the stakeholder survey component of the environmental scan, designed to produce an inventory of existing organizational supports for KT, along with general information about perceived challenges, facilitators and effectiveness of the supports. The scoping literature search that preceded the survey identified the absence of national surveys globally on this topic and informed the design of the survey. Follow-up qualitative interviews with organizations from the survey phase reporting established KT support units, the findings of which will be reported in a subsequent publication, gathered more detailed information about funding sources, models of service delivery, governance structures, characteristics and training of KT support personnel, evaluation methods, persisting KT support needs and barriers to KT support provision. Together, these insights are critical for advancing organizational evidence use and for enhancing research relevance in child health.

## Methods

The SQUIRE 2.0 checklist [18] was used to guide reporting of this environmental scan. Because of its nature as a quality improvement/programme evaluation initiative, our university-health centre’s joint research ethics board deemed ethics approval unnecessary.

### Survey development

The survey consisted of single- and multiple-response option questions, Likert scales and free text items. Survey items were developed based on analysis of an existing survey protocol proposed for adult health care organizations [10]. Additional items were developed to gather data about challenges and successes, primary recipients of supports, funding, human resources and roles, internal and external collaborations, stakeholder engagement in research and specific supports across the spectrum of the EIHC and KT processes. Supports deemed to be of interest to our team included, but were not limited to, personnel, resources, services, organizational structures or processes and/or internal or external partnerships/collaborations. The aims of the supports of interest could include providing access to research evidence, facilitating its sharing, supporting the appraisal, adaptation or implementation of evidence in practice or policy, promoting the evaluation of evidence use in health care or research, and/or building individual and/or organizational capacity for EIHC or KT. Respondents’ demographic information was also gathered with respect to role, department and years with the organization. Data on organizational characteristics, such as type (i.e. research versus health care), membership/staff size and geographic separation of the research institute and the health care organization, were also collected.

The survey draft underwent internal review by two senior leaders of KT within our organization. The refined version was translated into French to enable participation in the two official languages of Canada. Additional file 1 presents the research questions alongside their corresponding survey item numbers. The complete survey is provided in Additional file 2.

### Inclusion/exclusion criteria

The survey targeted leaders at paediatric AHSCs and their affiliated research institutes as the key stakeholders. AHSCs are healthcare organizations with missions involving not only excellence in the delivery of clinical care, but also with strong research and education mandates. These organizations typically maintain affiliations with academic institutions [19] and may have affiliated research institutes. Inpatient and ambulatory primary care settings and rehabilitation settings were eligible for inclusion. Children’s Healthcare Canada (formerly the Canadian Association of Paediatric Health Centres [CAPHC]) assisted in identifying the eighteen health care organizations and their seventeen research institutes across eight provinces for inclusion. Targeted stakeholders included directors of clinical care and research institutes, and leaders of clinical programmes, professional practice, EIHC/KT support units, libraries, education, quality improvement and grant facilitation offices. Other individuals (e.g. clinicians, KT support staff) with knowledge of the topics under study were also free to respond. These criteria excluded smaller and typically lower resourced paediatric health care organizations, such as child development centres, smaller rehabilitation centres, regional health units, schools, private practice therapy providers, infant development programmes and outreach teams. Larger AHSCs would be more likely to have implemented a broader range of KT supports as compared to these community-based or regional services.

### Recruitment

Participants indicated consent before accessing the survey. Prospective respondents were invited to participate through librarian listservs, social media and blog posts, emails distributed through project team members’ professional networks, the Canadian Child Health Clinician Scientist Program (CCHCSP) mailing list targeting child health clinician-researchers, and the CAPHC mailing list and a webinar presentation that targeted health administrators. Snowball sampling followed through invitations to individuals identified by survey respondents as having knowledge of the topic and roles at qualifying organizations. Up to two reminders were sent to prospective participants.

### Data collection and analysis

Data were collected and managed using REDCap electronic data capture tools hosted at our organization’s research institute [20]. Data provided by multiple respondents about a given organization were compiled for analysis at the organizational level. Generally, responses indicating the presence of a given support were taken to represent the organizational context in instances in which two respondents provided conflicting responses. When discrepancies existed between respondents, respondent roles were used to determine the most credible source (e.g. librarian, for information about library services). Data about EIHC/KT supports were grouped thematically and organized in accordance with the AIMD Framework [21]. This framework was chosen because it was designed and validated to describe interventions (in this case, organizational supports) to promote and integrate evidence into health care [21]. The framework’s four components operationalize the Aims, key active Ingredients, Mechanisms of action and Delivery methods of KT interventions [21]. Mechanisms of action categories were drawn from the Behaviour Change Wheel Framework. These categories specify the means by which the active ingredients are presumed to produce the aim [22]. The nine categories are Education, Persuasion, Incentivization, Coercion, Training, Enablement, Modeling, Environmental Restructuring, and Restrictions [22]. Knowledge of the AIMD components of existing KT strategies can assist in prioritizing the selection of appropriate supports for a given organization based on its goals, available resources and identified barriers to KT. Barriers were grouped thematically and presented in narrative form. Descriptive statistics, including frequency counts and proportions, were calculated for multiple-response option questions and demographics.

## Results

### Demographics

Thirty-one respondents from 17 of 35 possible sites (49%) participated in the survey. These sites included nine health care organizations, seven research institutes and one organization funded by the provincial health authority, with a mandate to support EIHC/KT at all of its health centres. In total, 78% (14 of 18 possible) health centre-research institute dyads (i.e. pairs) were represented. Snowball sampling contributed to 19% [6] of respondent recruitment. Respondents reported a mean of 12.6 (range 0.75 to 33) years with their organization. Respondents’ diverse positions included director, manager, knowledge broker, research development coordinator, professor, programme officer/coordinator, clinician, researcher, professional or collaborative practice lead, healthcare improvement specialist and librarian. Respondents selectively completed the sections of the survey (e.g. library services, stakeholder engagement) which they perceived having knowledge about their organization’s supports.

Represented provinces included Alberta (5 sites), Ontario (4 sites), British Columbia (3 sites), Quebec (2 sites), Manitoba (1 site), Nova Scotia (2 sites) and Newfoundland and Labrador (1 site). Saskatchewan was the only province with a paediatric AHSC that was not represented. Organization size ranged from 200 to 3700 staff and researchers. Ten of the 14 dyads (71.4%) reported sharing a physical site.

### EIHC/KT supports

**Fig. 1:**
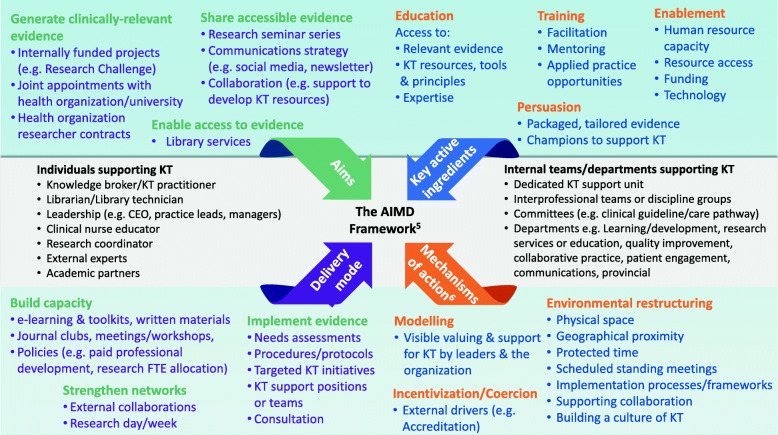
Summary of reported organizational supports for EIHC/KT, detailed according to the AIMD framework [21]. Note: EIHC = evidence-informed health care; KT = knowledge translation; FTE = full-time equivalent; CEO=Chief Executive Officer

**Fig. 2:**
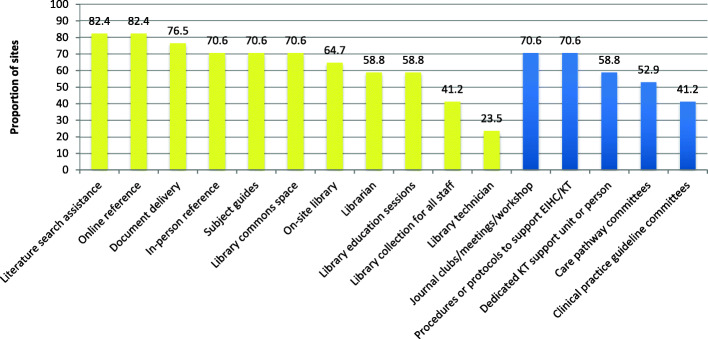
Proportion of sites reporting specific organizational supports. Note: Library services shown in yellow; other survey-prompted supports in blue (shaded)

Five sites reported having a dedicated staff person to support KT/EIHC, and six sites reported having a KT support unit or team. The organizational supports reported for EIHC/KT are summarized in Fig. 1 in accordance with the AIMD Framework [21]. Included in the figure are the objectives, target audiences, theorized key active ingredients, mechanisms of action and delivery methods for the supports. Eight of the nine mechanisms of action from the Behaviour Change Wheel were represented, with Incentivization represented only by external sources (i.e. Accreditation). Missing was the Restrictions category, which relates to the use of rules to decrease or increase behaviours [22]. An example of a support that would fall under this category is the organization identifying best practices for a given condition, and reducing the use of other treatment approaches by requiring therapists to complete screening checklists that necessitate trying best practices before allowing alternative treatments. Additional details about the supports are summarized below. The proportion of sites reporting specific supports is presented in Fig. 2 (*n* = 17 sites).

Sixty-five percent (11) of sites reported having an on-site library. Funding for library services came from the health care organization (7 sites), a university library (2 sites) or the research institute (1 site). Mean full-time equivalent for librarians was 1.3 (range 0.3 to 3.0), and for librarian technicians, 1.5 (range 1.0 to 2.0). Mean full-time equivalent per 100 staff was 0.28 for librarians (*n* = 5) and 0.13 for library technicians (*n* = 3). Additional library services identified through open-ended responses included bibliometrics and support with evidence synthesis.

Library education topics included the following: formulating PICO (Population, Intervention, Comparison, Outcomes) questions, conducting systematic and scoping reviews (including design and analysis), creating other evidence syntheses, searching literature databases and grey literature, using citation management tools, developing evidence surveillance strategies (e.g. journal alerts), conducting environmental scans and tracking metrics on research impact. Formats included workshops, webinars, online resources and individual training/consultation.

Other educational opportunities were delivered internally or sought through affiliated research institutes and research groups, provincial, health or research organizations and provincial and national associations (e.g. Strategy for Patient-Oriented Research Support Units, Centre for Clinical Epidemiology and Evaluation, Centre for Health Evaluation and Outcome Sciences, Centre for Health Services and Policy Research, Centre for Healthcare Innovation Manitoba, CAPHC). These educational forums included Telehealth video broadcasts, webinars, workshops and courses, including formal KT/implementation science training (e.g. St. Michael’s Hospital’s KT Program; SickKids Hospital’s KT Professional Certificate).

Procedures or protocols to support EIHC/KT included developing or adapting clinical practice guidelines, carrying out the steps of evidence-informed practice, creating a KT plan and conducting an environmental scan or a rapid evidence review. Care pathway committees were described in some cases as department-specific (e.g. nursing).

Research supports were accessed from affiliated research institutes, partnerships with provincial SPOR Units, mentorship from academic partners and affiliated university libraries. Research and KT supports were also obtained from government-funded (e.g. Canadian Agency for Drugs and Technologies in Health, Alberta Innovates) or university-affiliated (e.g. CanChild, Health Research Methods, Evidence, and Impact, Alberta Research Centre for Health Evidence) organizations.

### Clinical research integration and stakeholder engagement

Respondents relayed an awareness of the benefits of integrating research within clinical sectors and engaging clinicians in research. Survey responses indicated that respondents recognized the need for formal supports and more investment in this area. Supports were delivered through specific departments, roles, funding, partnerships and organizational processes or programmes, some of which were contingent on external funding. In some cases, supports were more limited for clinical staff as compared to researchers. Table 1 outlines the supports for research-clinical integration, which included operational, human resource, capacity building and collaboration-related provisions. Strategies currently in place at one or more sites are marked with an ^a^; all other table entries reflect additional strategies or supports identified by respondents as being required, but not yet implemented.

**Table 1:** Supports for clinical research integration and stakeholder engagement in research

Operational	• Financial support to displace clinical or clinical support personnel (i.e. protected time for research) • Protected time for research support tasks (e.g. recruitment, coordinating participant visits, obtaining consent, collecting data/specimens) • Expediting research agreements within the hospital • Research-dedicated space on clinical units • More timely access to population-based health information • ^a^Incorporating research integration strategies into strategic objectives • Establishing knowledge translation support roles within clinical programme and committee structures • ^a^Research full-time equivalent allocation programme for clinical staff • ^a^Funding for projects • ^a^Funding for clinician-scientist training • Technology (e.g. online meetings and communication platforms)
Human resources	• Research proposal writing • Research methodology consultation • Data input and management support • Developing systematic reviews, meta-analyses and clinical guidelines • Information technology • Conducting research • Facilitation of research implementation in clinical sectors • Needs assessment to guide the enactment of targeted supports
Capacity building	• Research methodology • Grant writing • Navigating the ethics application process • Scientific writing • Knowledge translation • Development of knowledge broker competency development pathways • Needs assessment to guide capacity building efforts
Collaboration	• ^a^Hospital recruitment of investigators, with support to develop/implement their research programmes • Engagement of research sector with clinical managers and operational leads • Formal collaboration of clinicians with the research institute to support interaction and exchange, planning for capacity building, and research • ^a^Engaging RI Executive Director on hospital Operations committee • ^a^Engaging management, leadership, families and the Foundation to review clinically situated research projects • ^a^Research institute partnerships with regional organizations funded by the health authority/university (e.g. Northern Alberta Clinical Trials and Research Centre) • ^a^Clinical sector partnerships with a university department

^a^Supports in place in at least one site; all others were reported as additional support needs that had not been implemented

Three respondents identified specific roles within their organizations that facilitated clinical engagement in research: (1) a Clinical Team Investigator, who supported clinical staff to have dedicated research time and research institute affiliation; (2) university-funded research coordinators who worked with clinical staff to conduct research; and (3) a KT practitioner who worked with the clinical director to encourage clinician involvement in research.

Funding was reported to support specific projects of direct clinical relevance, which in some cases required clinician-researcher collaborations. Some projects were funded, facilitated or overseen by an internal hospital department. Moreover, some research institutes had dedicated funding for clinical research capacity building, clinician-scientist training (in partnership with the CCHCSP), clinician-community research integration and support, resident research projects or KT/implementation grants to support clinical staff involvement in projects or to mobilize clinical practice change. Funding came from internal (including hospital-affiliated charitable foundations) or external sources.

**Fig. 3:**
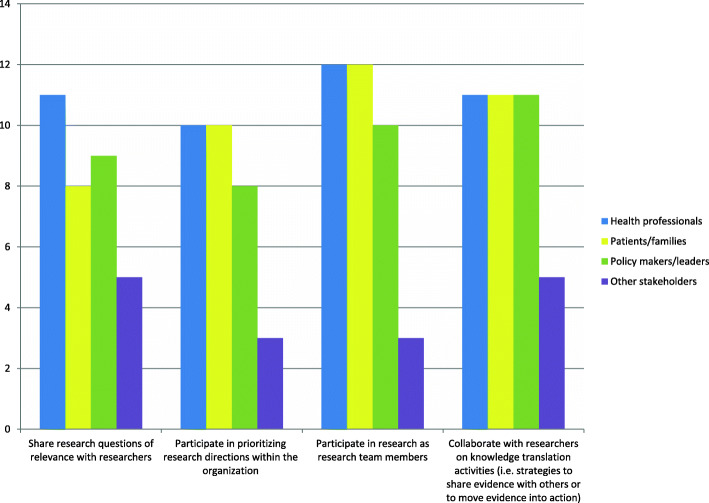
Frequency of site -reported supports for stakeholder engagement in research, by group (*n* = 17)

Fifteen sites (88%) reported formal collaborations between internal researchers and clinicians to conduct primary research and/or systematic reviews, while 12 (71%) reported the same for external researcher-clinician collaborations. However, approximately one third (6, 35% and 7, 41%, respectively) reported only occasional collaboration in these areas. Formal invitations or forums for researchers to share evidence with the organization’s clinical sector were reported at 16 sites (94%), with 12 (71%) and 10 (59%) sites reporting regular, frequent or continual activity in this area for internal and external researchers, respectively. The frequency of reporting of supports to engage these and other stakeholder groups in various research or KT phases is presented in Fig. 3.

Clinicians external to the AHSCs were engaged in EIHC/KT through networks (e.g. with other health organizations or national associations), partnerships (e.g. with health authorities or universities) and collaborations (e.g. with researchers) or through the organization’s funded research initiatives (e.g. a Research Challenge programme for staff). Logistical engagement strategies included tele/videoconference applications, the selection of physical meeting places to mitigate geographical barriers and outreach education by clinicians in rural areas, during which they shared evidence with and mentored community-based clinicians. Offering professional development opportunities to external clinicians, support from a KT practitioner or clinical research unit consultant on request, maintaining a resource website developed for community stakeholders, and tailoring project-specific KT materials to a specific site, stakeholder group or network were other reported methods. One site relied on external organizations (e.g. SPOR Unit) to carry out this work.

### Perceived effectiveness of EIHC/KT supports

Eleven (35.1%) participants responded to this section. Personnel supports perceived by respondents as being most effective in supporting EIHC/KT were staff identified as champions to support KT, and establishing a dedicated KT practitioner, committee or quality improvement/KT support unit with KT expertise and protected time. Activities linked to these roles included creating resources, collaborating or partnering with clinical sectors around evidence-informed standards of care, developing a KT strategy and building capacity. Research support personnel for clinicians were also identified as a facilitator for EIHC/KT.

Several specific KT support initiatives or programmes were distinguished as being most effective. Multiple sites mentioned library services. A rapid review service was described, which supported clinical leaders to access and apply evidence to inform decision-making. A knowledge broker initiative involved clinicians acting as knowledge brokers to facilitate evidence use within their clinical sectors. A student evidence-informed practice initiative was developed to build capacity for students and clinicians and to increase awareness of clinically relevant evidence to apply to practice. A Research Challenge programme was also established to support researcher-clinical collaborations in clinically relevant research. A structured continuous quality improvement management system, and site-wide projects supported visibly by sponsors and marketed in a way that resonated with clinicians were also seen as facilitators. Formal support infrastructure (e.g. dedicated team or personnel, resourced initiatives) was favoured over informal supports (e.g. relying on personal networks, one-time project assistance).

Respondents touted the merits of support and commitment for research, KT and innovation from leadership (e.g. Chief Executive Officer, interprofessional practice chiefs, managers). Inter-departmental (e.g. public relations, clinical practice and education, project management office, research institute) supports, including those for capacity development, imparting research expertise and KT support, as well as mentoring and external drivers for engaging in KT (e.g. Accreditation Canada, Best Practice Spotlight Organization candidacy) were also perceived as effective. Respondents also emphasized the importance of tailored, contextually relevant KT products, such as clinical toolkits, or evidence syntheses.

Logistically, protected time, close proximity of research and clinical staff, scheduling standing meetings to facilitate interaction, funding (including for strategic flagship projects), and greater collaboration between clinical and research programmes or teams, and between the KT support unit and clinical leadership, were also perceived as enablers of EIHC/KT. One respondent also identified the value of provincial practice leads that disseminate knowledge and clinical practice information as an effective support for KT.

### Barriers to supporting EIHC/KT

Operational constraints, such as caseload demands, limited clinician time or capacity to backfill, and competing priorities, were perceived to limit the delivery of EIHC/KT supports. Lack of resources, including funding, personnel and research support, were also identified barriers. Individual-level barriers included lack of research expertise within clinical sectors, skills across the entire spectrum of the KT process, clinician interest in engaging in research and/or EIHC/KT and awareness of existing supports. The state of the evidence in paediatric rehabilitation and the tendency not to share local solutions with the larger health care community were also raised as limiting factors.

The lack of a strong, overarching approach for KT support (e.g. only a single KT practitioner holding other concurrent roles) was seen as cause for lack of success. At times, the success of the supports was unclear, as exemplified by one respondent’s statement: “I’m not sure that any of our [organization’s] strategies have been particularly effective.” At one site, capacity building was a challenge because of staff turnover and lack of consistency in training new staff. KT support personnel also lacked practice opportunities to sustain their skills. Challenges in identifying personnel with training and practical experience in KT support existed for those hiring. Supports and networks for stakeholder engagement were seen as poorly established, and sustainability of KT support infrastructure was perceived to need greater attention. Finally, one respondent indicated the need to focus efforts on fostering a culture that values KT and prioritizes EIHC.

The most significant barriers perceived by respondents were lack of funding, time, personnel, management support, understanding about the processes of care, knowledge about research, KT and implementation and how to facilitate it, and limited staff capacity to support and to engage in change. Themes relating to barriers in communication (e.g. inability to connect with staff because of its size or email response habits), and silos between clinical teams, research and education departments also predominated.

## Discussion

This survey provides a snapshot of the many organizational EIHC/KT supports that exist within paediatric AHSCs and their affiliated research institutes in Canada. Existing studies and single-organization case studies typically report a single or few strategies (e.g. resource development and training; creating syntheses; sharing evidence; fostering collaborations; use of KT theory) [23, 24]. In contrast, this environmental scan identified and synthesized an extensive range of organizational structures, processes, personnel and partnerships. Although the use of many of these strategies has been confirmed in other sectors (e.g. public health, education, military health, universities) [23, 25], the diversity of service models presented here highlights the need for greater evidence to inform best practices, resource allocation and decision-making in this area.

### The sample

Our response rate represented 49% of our target sites and exceeded the mean organizational survey response rate in the literature of 35% [26]. The balance of research and health organizations provided strong representation from both institution types. Representation from the majority of provinces supports the generalizability of the findings. The diverse leadership and professional roles, and experience of respondents lends credibility to their reporting. Recruiting multiple respondents at eight sites provided greater comprehensiveness in the reporting. Although the sample was limited to AHSCs, some of these KT supports may be appropriate in other health care contexts (e.g. KT needs assessments; external collaborations; accessing external seminars; professional development/training; champions; use of implementation processes and frameworks; building a culture of KT; modeling by leadership). Indeed, similar approaches (e.g. partnerships, leadership, training, structured processes) have been documented within the United Kingdom’s CLAHRC model to support researcher-clinician collaboration in research [27–31]. However, feasibility and cost-effectiveness for a smaller staff size or resource availability may limit capacity for implementation, or the appropriateness or potential for impact of some types of supports (e.g. joint researcher appointments; internally funded research projects; research week; funded KT support personnel or teams; physical space). External sources of KT support may be most relevant for these latter types of strategies. This environmental scan provides a starting point for such organizations to seek out these sources and to explore their value to internal strategic objectives and staff needs.

### Supports

The limited access to library collections, especially for those without university affiliations, has significant implications for accessing and applying current research. Ellen and colleagues identified library access as a priority for enhancing evidence use in health care organizations [8]. The predominant funding burden left to health care organizations for library services has the potential to draw resources from other operational priorities, or to result in the organization’s information access needs not being met. Additional work is thus required to determine the extent to which library staffing levels meet organizational needs.

The environmental scan revealed a greater number of dedicated KT support units than anticipated. However, for other sites, these responsibilities being left to one or two individuals, or being informal and unfunded leaves greater potential for inconsistent or inadequate coverage. Apart from the supports specifically prompted on the survey, little overlap existed in responses between participants, which spanned nearly all of the Behaviour Change Wheel mechanisms of action [22]. This observation reinforces the diversity and inconsistency of supports. The range of external organizations from which KT supports were drawn may reflect the differing support landscapes across provinces. Further detail gleaned from our follow-up interviews will provide insight into the structures, services, personnel, training methods and governance of these support units, and the availability of internal and external resources. In-depth examination of each support and its proposed mechanisms from the perspectives of those delivering the supports will generate a clearer picture of their key ingredients, mechanisms of action, and delivery modes. Implementing all of the reported EIHC/KT supports would not be feasible. However, having a snapshot of different models of support can help health and research administrators identify strategies by which to assess or to establish their own support infrastructures.

### Integrating research and stakeholders

Despite their three-pronged mandate for clinical care, research and education [32], variable supports existed for research integration in clinical settings across these AHSCs. Although research integration within clinical settings themselves is not a requirement of this mandate, such an approach, which engages clinician end-users as research partners, has the potential to improve organizational performance [33], enhance the relevance of the research, and facilitate its implementation in practice or policy [34]. Examples of such models exist (e.g. [35–37]), which may involve researchers embedded in clinical sectors [38], co-funded research [38], support for implementation research [37], introducing structured processes to identify and to address gaps in best practices through research activities [37], establishing communities of practice to address these gaps [36], and the development of new roles and core units to support strategic clinical research integration and KT [37]. From a leadership perspective, these strategies may involve a shared governance structure across health care and research organizations [38], distributed leadership across different stakeholder groups [28], stakeholder-engaged advisory committees [37] and senior leadership, research and management positions held, or initiatives led, by individuals with both health care and academic appointments [36, 38]. Through these modes, research directions can be aligned with health care priorities, and findings can be implemented more efficiently while building research and KT capacity for both clinicians and researchers [38].

The operational, human resources, capacity building activities and collaboration/network building to support research integration were similar in nature to those for EIHC/KT; using similar infrastructure may facilitate implementation. Given that the majority of identified barriers to clinical research integration related to engaging the clinical sector, this area represents a priority for supporting implementation. More information is required from the clinical sector to determine the extent to which the current leadership and research roles, project funding programmes, full-time equivalent (FTE) allocation programmes, external partnerships, and hiring of hospital-funded investigators are effective at mitigating these barriers. Structured context-specific needs assessments are required to understand more clearly the challenges and opportunities for strengthening clinical research integration in order to inform the implementation of these recommendations.

Broader networks seemed to be the primary strategy for engaging external clinicians in EIHC/KT, although internally funded projects and professional development opportunities were also offered. The extent to which external clinician involvement is prioritized as a mandate for organizations may positively influence these efforts. For example, health care organizations with a responsibility to support community-based health professionals may be required to report on engagement outcomes and therefore to establish and to maintain infrastructure that sustain these outcomes. These efforts might be conducted through resource websites, outreach processes, consultation services, and so forth.

Supports for stakeholder engagement were less common than for other aims (i.e. KT and clinical research integration), and room for improvement exists. Caution should be taken in interpreting data related to the frequency and pervasiveness of stakeholder engagement, as it may not reflect engagement rates at the research project level accurately.

### Evaluation

The smaller proportion of respondents that reported on the effectiveness of EIHC/KT supports may reflect a lack of knowledge on the part of respondents, or a lack of evaluation and reporting practices related to these services. Respondents mentioned no specific outcomes or indicators, and supporting effectiveness data was not requested. More detailed data about reported evaluation processes and outcomes during the interview phase will provide more objective reports of effectiveness, and information by which to inform best practices in the evaluation of EIHC/KT supports.

The broad range of audiences for KT supports makes their provision more complex, as different stakeholders require different supports, and messaging, terminology and delivery must be tailored. However, many of these supports may benefit different groups simultaneously, such as highly trained personnel, targeted initiatives, leadership, interdepartmental expertise, external drivers and logistical support. Detailed description of these initiatives perceived as most effective may allow others to learn more about their features and mechanisms of action, and to adapt, apply and evaluate them elsewhere.

While having a dedicated KT practitioner was mentioned as the most effective support by one respondent, more research is needed to determine adequate staffing levels for KT support personnel, given the negative impact of limited reach by smaller support teams. A needs assessment that takes into account capacity and resources can help to inform the development of a targeted, context-specific EIHC/KT support plan that integrates leadership, personnel, network building, logistical processes and infrastructure with structured programmes, support roles, and resources as appropriate.

### Barriers

Clearly, additional resources were seen as key to the success of EIHC/KT supports, along with a coordinated approach involving more than a single KT practitioner. Limited supports may compromise capacity to support all staff, leading to inequities. Lack of research expertise within clinical sectors, and lack of skills across the entire spectrum of the KT process, point to the need for additional supports for capacity building, consultation and/or facilitation. However, effective communication about the nature of supports available and who is eligible to access these supports may support broader reach and impact of services. Systematic data collection can yield insights into the reach, satisfaction, effectiveness and impacts of EIHC/KT supports.

Sustainability also appears to require ongoing education and training, not only for KT support personnel, but also for staff, to address workforce turnover. Structured external KT training programmes may help to address this gap in the absence of tailored internal programmes. Knowledge of the health care and research contexts, and a bridging of their cultures may also be necessary to facilitate effective collaboration and research integration. One support strategy alone is not considered adequate to enable real change [9]. However, limited funding exists in the Canadian health care system for these non-clinical priorities. External partnerships, strategic use of research and quality improvement resources and less resource-dependent strategies (e.g. strengthened communication networks, establishing protocols and forums to support research and KT processes, leadership commitment) may be important for sustainability and for strengthening a culture of research and EIHC/KT.

### Limitations

The AIMD Framework was used in the analysis phase but not to design the survey items. As a result, some detail about the aims, ingredients and mechanisms of action of the KT supports was missing, making it unfeasible to document each element of the AIMD Framework for each support identified. Instead, characteristics of all of the supports were grouped under each component heading. Further exploration into the characteristics of these supports, particularly in the context of empirical studies, would be beneficial in defining them further, and in evaluating their effectiveness at achieving their intended aims. This environmental scan and its analyses provide a foundation for this work.

In some instances, the survey yielded brief answers. As a result, we may not have captured details, context and layers of qualitative richness [39], leading to the potential for misinterpretation or misrepresentation of the respondent’s intent and in some cases, limited information related to funding, perceived strengths, and evaluation methods. Our in-depth follow-up interviews that can probe for context, personal meaning, emotional and social nuances [39] will address this gap and elicit further detail. Respondents also shared their organizations’ support strategies and challenges with the understanding that confidentiality would be maintained during reporting. As a result, readers who desire more information about funding and specific EIHC/KT support strategies lack the ability to make direct contact with site representatives to learn more. Joining provincial or national networks focused on KT may provide a forum for continuing the conversation and connecting those individuals or sites.

### Future directions

Future directions include comparative research, for example through multi-site or multi-programme studies that employ realist evaluation [40], to determine which supports are effective in which contexts and at what intensities [8], and economic analysis to determine the cost-effectiveness of these supports. The results of this survey will also inform the development of the interview guide to examine in greater depth the structure, personnel characteristics, resources and sustainability of the dedicated KT support units identified through the survey. Comprehensive study of individual sites may also enable a clearer understanding of the multi-faceted factors that influence EIHC/KT support from the perspectives of health care professionals, leaders, researchers and KT support personnel. No studies on whole systems of supports within an organization currently exist [41]. A systematic approach that describes the different KT support unit models of support is critical. This exploration should aim to capture the interaction between organizational supports and individual-, network- and systems-level barriers and facilitators of EIHC/KT in order to inform tailored strategies to optimize evidence use within these organizations.

## Conclusions

This environmental scan summarizes for the first time the supports for EIHC/KT, clinical research integration and stakeholder engagement in place within paediatric research and health care organizations across Canada. Serving up to nine different stakeholder groups, the supports leveraged individuals, internal departments and external organizations to facilitate the co-production of research, access to and implementation of evidence, capacity building, evaluation and network strengthening. These novel findings can be used to inform the design or refinement of EIHC/KT support programmes within AHSCs and their affiliated research institutes. Following a local assessment of the barriers and facilitators of evidence use, targeted strategies can be considered from the array of approaches described. Deliberate attention to the intended mechanisms of action and key ingredients of the support strategies can ensure that appropriate modes of delivery are enacted. Evaluation in practice will provide greater insight into the effectiveness and sustainability of these supports. No one-size-fits-all solution exists for supporting EIHC/KT. Organizational objectives and needs, internal and external resources, and training and partnership opportunities will influence the directions taken by those facilitating EIHC/KT, research integration and collaboration. The findings provide an important foundation for enabling research use from an organizational perspective, and for supporting the involvement of clinical and other stakeholders to augment the relevance and impact of child health research.

## Supplementary Information

**Additional file 1.** Survey development and research questions. Table of research questions and their corresponding survey items that guided survey development.

**Additional file 2.** Survey items. Complete survey administered to participants.

## Data Availability

The datasets generated and analysed during the current study are not publicly available to protect the privacy and confidentiality of individual respondents whose identities may be discernable from their responses, but are available from the corresponding author on reasonable request.

## Abbreviations

EIHC: Evidence-informed health care
KT: Knowledge translation
AIMD: Aims, Ingredients, Mechanism, Delivery
AHSC: Academic health science centre
CAPHC: Canadian Association of Pediatric Health Centres (recently renamed Children’s Healthcare Canada)
CCHCSP: Canadian Child Health Clinician Scientist Program
PICO: Population, Intervention, Comparison, Outcome
FTE: Full-time equivalent
CEO: Chief Executive Officer
ARCHE: Alberta Research Centre for Health Evidence
SPOR: Strategy for Patient-Oriented Research
CADTH: Canadian Agency for Drugs and Technologies in Health
OACRS: Ontario Association of Children’s Rehabilitation Services
