# Supplementary material for: Organizational supports for knowledge translation in paediatric health centres and research institutes: insights from a Canadian environmental scan
Source: Implement Sci Commun. 2021 May 13;2:49. doi: 10.1186/s43058-021-00152-7 (PMC8117660; doi:10.1186/s43058-021-00152-7)
Supplement: Supplementary file 1 — Additional file 1. Survey development and research questions. Table of research questions and their corresponding survey items that guided survey development. [file 43058_2021_152_MOESM1_ESM.docx]

**Additional file 1. Survey development and research questions**

| **Research Questions** | **Data Collected** | **Question #** |
| --- | --- | --- |
| What are the organizational and demographic characteristics of the respondents? | - Type of organization - Size of organization - Titles, roles of participants - Physical proximity of research & clinical organizations | 1-9 |
| What personnel supports are in place to support EIHC/KT? | - Number of EIHC/KT support personnel - Role of EIHC/KT support personnel | 10b, 13 |
| What resources exist to support EIHC/KT? | - Funding structure/source for EIHC/KT support - Internal resources used to support EIHC/KT - External resources used to support EIHC/KT | 10-13, 15 |
| What EIHC/KT services are provided by the organization? | - Educational opportunities for EIHC/KT capacity building - Services or supports available for evidence access, appraisal, adaptation, synthesis, sharing, implementation or evaluation | 10-13 |
| What organizational structure or processes exist to support EIHC/KT? | - E.g. Support for research integration into clinical programs, forums for sharing research, processes for adapting clinical practice guidelines or identifying the need for behaviour change, etc. | 13 |
| Who are the primary audiences for these organizational supports? | - Direct recipients of identified supports | 14 |
| What are the primary internal and external partnerships, collaborations or linkages that facilitate EIHC/KT? | - Internal collaborations or linkages to support EIHC/KT - External partnerships or collaborations to support EIHC/KT | 16-18 |
| Which supports have been most successful in facilitating EIHC/KT? | - Successes and facilitators identified | 19, 22 |
| What are the challenges associated with facilitating EIHC/KT at the organization? | - Challenges or barriers identified, including resource constraints, individual, organizational and system-level barriers | 20, 21 |

Note: EIHC=evidence-informed health care; KT=knowledge translation
