# Supplementary material for: Organizational supports for knowledge translation in paediatric health centres and research institutes: insights from a Canadian environmental scan
Source: Implement Sci Commun. 2021 May 13;2:49. doi: 10.1186/s43058-021-00152-7 (PMC8117660; doi:10.1186/s43058-021-00152-7)
Supplement: Supplementary file 2 — Additional file 2. Survey items. Complete survey administered to participants. [file 43058_2021_152_MOESM2_ESM.docx]

**Additional file 2. Survey items**

Thank you for participating in this Environmental Scan being conducted by staff at Sunny Hill Health Centre for Children in Vancouver. Our aim is to understand the barriers and supports that exist within pediatric academic health science centres and their research institutes, with respect to evidence informed health care (EIHC) and knowledge translation (KT).

**Definitions:** EIHC and KT refer to the processes of moving evidence into action in health care. Evidence refers to research findings, as well as professional experience and patient/family perspectives, which are considered together in making informed decisions.

**EIHC** is typically carried out by health care providers, health leaders and policy makers during the decision-making and implementation processes involved in developing, delivering and making changes in health services. **KT** is usually understood to reflect the process carried out or facilitated by researchers and others to identify the need for evidence, to support the effective adapting/packaging and sharing of evidence, to identify and apply strategies that target barriers to implementation, and to evaluate evidence use in health care.

If you are not familiar enough with the specific EIHC/KT supports being addressed in a particular section of the survey, please feel free to provide the name and contact information of a colleague who may be able to respond in more detail.

**DEMOGRAPHICS**

**Your Organization**

1. Please select your primary organization from the drop-down menu, or start typing your response in the text box: [check one – text auto-completes]
   1. [All organizations listed here]
2. Please select your secondary organization from the drop-down menu, or start typing your response in the text box: (check one – text auto-completes)
   1. List all the orgs here
3. Please list any other organization(s) with which you are affiliated (if applicable):

**About you:**

1. What is the name of your home department/unit/sector at your primary organization?
2. What is your role/title within the organization? {Responses will be grouped in any reports of the survey)
3. How long have you been at the organization? ____________________
4. With respect to my primary organization, I am knowledgeable about the following topics (check all that apply): (Check the topics about which you would like to respond on the survey)
   1. The number of staff/members
   2. Library services
   3. Other (non-library resources, personnel and services in place to support EIHC/KT
   4. Research/clinical collaboration and/or supports for research within clinical sectors
   5. Effectiveness of EIHC/KT supports
5. Please provide the email addresses of individuals at your organization with knowledge about these topics so that we may invite them to take the survey (optional):

**-------------------------------------------------------pAGE BREAK-------------------------------------------------------**

**STAFFING AND MEMBERSHIP**

1. How many health professionals are employed at your organization?
2. How many researchers are affiliated with your organization?
3. Do the research institute and the clinical sector share the same physical site? (yes/no/unsure)

**-------------------------------------------------------pAGE BREAK-------------------------------------------------------**

**LIBRARY SERVICES**

1. Does your organization have a library onsite? (yes/no/unsure)
   1. If no, is there a digital library or electronic resources available to staff online? Yes/no/unsure
   2. If yes, how is it staffed?
      1. No staff
      2. Librarians
         1. How many full-time equivalent (FTE) librarians?
      3. Library Technicians
   3. How many full-time equivalent library technicians?
      1. Unsure
      2. Other (please describe): ______________
         1. You selected ‘other’ – please describe staffing, including job title(s) and full-time equivalent (FTE) staff numbers):
   4. If yes, how are the library and its services funded? (Check all that apply)?
      1. Branch of the university library
      2. Your health care organization
      3. Your research institute
      4. Unsure
      5. Other (please describe): ______________
2. My organization is affiliated with a University library (yes/no/unsure)
3. If yes, does the collection serve all hospital staff whether or not they have a formal University appointment (i.e. Affiliated status)? (yes/no/unsure)
4. If no, does your organization provide a library collection (digital and/or print) for staff who are not affiliated with the University? (yes/no/unsure)
5. Which of the following library services are offered to all staff? Please check all that apply:
6. Document Delivery and/or Interlibrary Loan
7. Online reference (e.g. Library staff available to help with locating materials, assistance with literature searching, etc.)
   1. In-person reference
8. Subject or resource guides
9. Online tutorials
10. Literature searching
11. Education and training
    1. Please describe the education/training offered:
12. Staffed library/learning commons space
13. Other
    1. You selected ‘other’ – please describe
14. Which department at your organization oversees library services?

*Please contact the following person(s), whose input/perspective would be valuable for this section (optional): ____ [Please enter an email address]*

**-------------------------------------------------------pAGE BREAK-------------------------------------------------------**

**NON-LIBRARY SUPPORTS FOR EIHC/KT**

1. Resources, services, processes, personnel and/or infrastructure are provided by my organization to support the following aspects of EIHC/KT:
   1. Accessing/acquiring research evidence (yes/no/unsure)
      1. If yes, please describe these supports, including the role/title/department of support personnel (if applicable):
   2. Assessing/appraising evidence for its level of evidence (i.e. study design), quality and [clinical] applicability AND/OR adapting, packaging evidence for specific audiences/stakeholders (e.g. rapid reviews, clinician-friendly summaries of evidence, synopses of systematic reviews, infographics, handouts/pamphlets, videos, etc.)
      1. If yes, please describe these supports, including the role/title/department of support personnel (if applicable):
   3. Sharing/disseminating evidence with key stakeholders (e.g. clinicians, patients, health leaders, researchers, etc.)
      1. If yes, please describe these supports, including the role/title/department of support personnel (if applicable):
   4. Identifying the need for, and/or assessing barriers to evidence use/behaviour change
      1. If yes, please describe these supports, including the role/title/department of support personnel (if applicable):
   5. Developing [practice/policy change or KT] plans for moving evidence into action
      1. If yes, please describe these supports, including the role/title/department of support personnel (if applicable):
   6. Evaluating evidence use e.g. monitoring implementation/scale-up efforts and outcomes
      1. If yes, please describe these supports, including the role/title/department of support personnel (if applicable):
   7. Capacity building (e.g. training/educational opportunities/developing resources or tools to support individual competency development in EIHC/KT and/or organizational capacity for EIHC/KT)
      1. If yes, please describe these supports, including the role/title/department of support personnel (if applicable):
2. The following other organizational structure or processes exist at my organization: (yes/no/unsure):
3. Departments/units/teams that support EIHC/KT
4. Clinical practice guideline committees
5. Care pathway committees
6. Procedures or protocols
7. Journal clubs, interactive meetings or workshops to share new research evidence with staff
   1. Other supports/resources
      1. Please describe these supports, including the role/title/department of support personnel (if applicable):

**EIHC/KT SUPPORT RECIPIENTS**

1. Who do your EIHC/KT personnel/resources support? i.e. what is the target audience for these supports? (Check all that apply)

- Health professionals
- Health professional students/residents
- Organizational leaders
- EIHC/KT support staff
- Researchers
- Research support staff
- Trainees
- Patients & families
- Knowledge brokers
- Other (please describe): ______

1. What activities to support EIHC/KT for your organization are provided by external personnel/organizations?
2. From which organization(s) do you access this support?

*Please contact the following person(s), whose input/perspective would be valuable for this section (optional): ____ [Please enter an email address]*

**-------------------------------------------------------pAGE BREAK-------------------------------------------------------**

**Partnerships AND collaboration**

1. How is research supported within clinical sectors/programs by your organization? e.g. what supports, resources and/or mechanisms exist to support research at the point of care?
2. What additional supports are needed?
3. How frequently does your organization do each of the following activities to produce evidence and/or move evidence into action? (never/occasionally/regularly/frequently/always/unsure)
4. Formal collaborations between internal researchers and clinicians to conduct primary research and/or systematic reviews
5. Formal collaborations with external researchers to conduct primary research and/or systematic reviews
6. Formal invitations to or forums for internal researchers to share evidence with the organization’s clinical sector
7. Formal invitations to or forums for external researchers to share evidence with the organization’s clinical sector
8. My organization has processes in place to engage the following stakeholder groups to… (yes/no/unsure/not applicable)
9. Share research questions of relevance with researchers
   1. Health care professionals
   2. Patients/families
   3. Policy makers/leaders
   4. Other stakeholders
      1. Which other stakeholders?
10. Participate in prioritizing research directions within the organization
11. Participate in research as research team members
12. Collaborate with researchers on knowledge translation activities (i.e. strategies to share evidence with others or to move evidence into action)
13. How is stakeholder engagement in research supported by your organization?
14. What additional supports are needed?
15. What supports/processes are in place to engage health professionals who are not on-site (whether internal or external to your organization) in EIHC/KT?

*Please contact the following person(s), whose input/perspective would be valuable for this section (optional): ____ [Please enter an email address]*

**-------------------------------------------------------pAGE BREAK-------------------------------------------------------**

**SUCCESSES & CHALLENGES**

1. Which supports do you think have been the most effective at supporting EIHC/KT within your organization?
2. Which supports do you think have been least effective at supporting EIHC within your organization?
3. What barriers or challenges limit the delivery of EIHC/KT supports at your organization?
   1. What is the most significant barrier to EIHC/KT support?
4. What factors have facilitated the delivery of EIHC/KT supports at your organization?

*Please contact the following person(s), whose input/perspective would be valuable for this section (optional): ____ [Please enter an email address]*

**-------------------------------------------------------pAGE BREAK-------------------------------------------------------**

**ADDITIONAL COMMENTS**

1. Please share any additional information about EIHC/KT supports for your organization that have not been addressed above: _______

**------------------------------------------------------pAGE BREAK--------------------------------------------------------**

Thank you for your time in completing the survey. We look forward to sharing the results with you!
